# Prognostic role of galectin-3 expression in patients with solid tumors: a meta-analysis of 36 eligible studies

**DOI:** 10.1186/s12935-018-0668-y

**Published:** 2018-11-03

**Authors:** Yi Wang, Shiwei Liu, Ye Tian, Yamin Wang, Qijie Zhang, Xiang Zhou, Xianghu Meng, Ninghong Song

**Affiliations:** Department of Urology, First Affiliated Hospital of Nanjing Medical University, No. 300 Guangzhou Road, Nanjing, 210029 Jiangsu China

**Keywords:** Prognostic role, Galectin-3, Cancer, Meta-analysis

## Abstract

**Background:**

Galectin-3 as a β-galactoside-binding protein, has been found to be involved in tumor cell growth, anti-apoptosis, adhesion, angiogenesis, invasion, and distant metastases, indicating that it may play a pivotal role in cancer development and progression. However, their results remain debatable and inconclusive. Hence, this meta-analysis was performed to clarify the precise predictive value of galectin-3 in various cancers.

**Methods:**

PubMed, Web of Science, Embase, Cochrane Library, CNKI and Wanfang databases were searched comprehensively for eligible studies up to July 15, 2018. Pooled hazard ratios (HRs) with 95% confidence intervals (CIs) of OS or DFS/PFS/RFS were calculated to demonstrate their associations.

**Results:**

A total of 36 relevant studies were ultimately enrolled in this meta-analysis. Our results shed light on the significant association of elevated galectin-3 expression with reduced OS or DFS/RFS/PFS in overall cancer patients (pooled HR = 1.79, 95% CI 1.42–2.27, *I*^2^= 67.3%, *p *< 0.01; pooled HR = 1.57, 95% CI 1.04–2.37, *I*^2^= 67.1%, *p *= 0.001). In tumor type subgroup analysis, we found high expression of galectin-3 was correlated with shorter OS or DFS/RFS/PFS in colorectal cancer (pooled HR = 3.05, 95% CI 2.13–4.35, *I*^2^= 0.0%, *p *= 0.734; pooled HR = 2.49, 95% CI 1.82–3.41, *I*^2^ = 0.0%, *p *= 0.738; respectively) and meanwhile it merely associated with reduced OS in ovarian cancer or non-small cell lung cancer (pooled HR = 2.24, 95% CI 1.38–3.64, *I*^2^= 0.0%, *p *= 0.910; pooled HR = 2.07, 95% CI 1.48–2.88, *I*^2^= 0.0%, *p *= 0.563; separately).

**Conclusions:**

Taken together, our results suggested that galectin-3 played an oncogenic role in colorectal cancer, ovarian cancer and non-small cell lung cancer, indicating it could be a promising biomarker and a novel therapeutic target for them. Further studies were warranted to validate our findings.

## Background

Galectins are a large family of widely distributed carbohydrate-binding proteins, characterized by their binding affinity for β-galactosides and conserved sequences in the binding site [1]. Meanwhile, galectins are often exhibited a high level of expression in cancer cells or cancer-associated stromal cells with the aggressiveness of tumors and the acquisition of the metastatic phenotype [2]. Because of their significant involvement in various biological functions and pathology, the role of galectins seems to be of importance [3]. Therein, galectin-3 also knew as LGALS3, L31, GAL3, MAC2, CBP35, GALBP and GALIG, belongs to the family of galectins [4]. In both extracellular and intracellular manners, galectin-3 exhibits its pleiotropic biological and molecular functions. Extracellularly, it has the ability to adjust microenvironment by means of interacting with the cell surface and extracellular matrix glycoproteins or glycolipids. Intracellularly, it was capable of modulating signaling pathways via interacting with cytoplasmic and nuclear proteins [5]. Up to now, a growing number of researches have suggested the involvement of galectin-3 in tumor progression and disease outcome [6–8].

Galectin-3 has been found to be differently expressed in various normal and malignant tissues. Previous studies indicated that down-regulation of galectin-3 was associated with loss of the transformed phenotypes in thyroid papillary carcinoma cells, but up-regulation of it could induce the transformed phenotype in normal thyroid follicular cell lines [9]. Accumulating data have demonstrated that different galectin-3 expression in tumor tissues was associated with unfavorable survival in cancer patients [10–14]. These studies concentrated on colorectal carcinoma, cervical carcinoma, breast cancers, gastric carcinoma, laryngeal squamous-cell carcinoma and so on. However, their results remained inconsistent. The discrepancies among these studies highlighted the importance of evaluating the prognostic significance of galectin-3 in multiple human malignant neoplasms. Hence, this meta-analysis was conducted to clarify the relationship between galectin-3 expression and the prognosis of patients with carcinoma. Last but not least, it is the first time for us to shed light on their relationship and galectin-3 is anticipated to be a prognostic marker in clinical applications.

## Materials and methods

### Literature search strategy

We conducted a comprehensive search of online databases PubMed, EMBASE and Web of Science, Cochrane Library, Chinese National Knowledge Infrastructure (CNKI) and Wanfang database (Chinese) to identify relevant literature published before July 15, 2018. The search strategy was mainly consisted of the following keywords in combination with Medical Subject Headings (MeSH) terms and text words: (“cancer” or “carcinoma” or “neoplasm” or “tumor” or “tumour”) and (“galectin-3” or “GAL3” or “LGALS3” or “L31” or “MAC2” or “CBP35” or “GALBP” or “GALIG”). In addition, potentially eligible articles were identified via meticulously searching from the reference lists of relevant reviews and original literature.

### Inclusion and exclusion criteria

The eligible studies needed to meet the following four inclusion criteria: (1) English or Chinese publications; (2) patients with carcinoma; (3) a relationship of galectin-3 expression with cancer prognosis; (4) sufficient data could be extracted. Additionally, the exclusion criteria included the following points: (1) non-English or non-Chinese research; (2) duplicates of the previous publication; (3) reviews or letters or case reports or comments or editorials; (4) unrelated to galectin-3 or human patients; (5) absence of key information.

### Quality assessment

The following information should be extracted from included articles before being evaluated: (1) the study population and country; (2) the study design; (3) assay method to determine galectin-3 expression; (4) the prognosis or survival assessment; (5) the detected tumor and pathology information; (6) the cutoff point of galectin-3; and (7) the follow-up duration. In addition, Newcastle–Ottawa Scale (NOS), as one of the most useful scale to evaluate the quality of non-randomized studies (http://www.ohri.ca/programs/clinical_epidemiology/oxford.htm), was independently evaluated by two blind reviewers [15]. The criteria of quality assessment were as follows: (1) representativeness of the exposed cohort; (2) selection of the non-exposed cohort; (3) ascertainment of exposure; (4) outcome of interest not present at start of study; (5) control for important factor or additional factor; (6) assessment of outcome; (7) follow-up long enough for outcomes to occur; (8) adequacy of follow up of cohorts. Total quality score of NOS was ranged from 0 to 9, which was regarded as high quality with the final score > 6. Details were presented in Table 1.

**Table 1:** Newcastle–Ottawa quality assessments scale

Studies	Year	Quality indicators from Newcastle–Ottawa Scale								Scores
		1	2	3	4	5	6	7	8	
Chou [20]	2018	★	–	★	★	★	★	★	★	7
Lu [10]	2017	★	★	★	★	★★	★	–	★	8
Huang [4]	2017	★	★	★	★	★	★	–	★	7
Li [11]	2017	★	★	★	★	★★	★	–	–	7
Shimura [41]	2017	★	★	★	★	★★	★	–	★	8
Wang [49]	2017	★	★	★	★	★	★	–	★	7
Liu [37]	2017	★	★	★	★	★★	★	–	–	7
Gopalan [21]	2016	★	★	★	★	★★	★	–	★	8
Ilmer [12]	2016	★	★	–	★	★★	★	–	★	7
Yang [48]	2016	★	★	★	★	★★	★	–	★	8
Tas [36]	2016	★	–	★		★★	★	–	★	7
Cheng [40]	2015	★	★	★	★	★★	★	★	–	7
Lu [47]	2015	★	★	★	★	★★	★	★	–	8
Jiang [22]	2014	★	★	★	★	★	★	★	★	8
Gomes [23]	2014	★	–	★	★	★★	★	★	★	8
Mu [44]	2013	★	★	★	★	★	★	–	★	7
Wu [45]	2013	★	★	★	★	★★	★	–	–	7
Liu [46]	2013	★	–	★	★	★	★	–	★	6
Yamaki [24]	2012	★	★	★	★	★★	★	★	–	8
Yang [25]	2012	★	–	★	★	★	–	–	★	5
Kim [26]	2012	★	★	★	★	★★	★	–	–	7
Kosacka [38]	2011	★	★	–	–	★★	★	★	–	6
Povegliano [27]	2010	★	★	★	★	★	★	–	★	7
Canesin [42]	2010	★	–	★	–	★★	★	–	★	6
Vereecken [43]	2009	★	★	★	–	★	★	–	★	6
Miranda [28]	2009	★	–	–	★	★★	★	★	–	6
Szoke [29]	2007	★	–	–	★	★	★	–	★	5
Kang [30]	2007	★	★	★	★	★	★	★	–	7
Moisa [39]	2007	★	★	–	★	★	–	★	★	6
Okada [13]	2006	★	★	★	★	★	★	–	★	7
Plzak [31]	2004	★	★	★	–	★	–	★	–	5
Piantelli [14]	2002	★	★	★	★	★★	★	★	–	8
Brule [32]	2000	★	–	★	★	★	★	★	–	6
Honjo [33]	2001	★	★	★	–	★	★	★	–	6
Nakamura [34]	1999	★	★	★	★	★★	★	–	★	8
Sanjuan [35]	1997	★	–	★	–	★	★	–	–	4

1. Representativeness of the exposed cohort; 2. Selection of the non-exposed cohort; 3. Ascertainment of exposure; 4. Outcome of interest not present at start of study; 5. Control for important factor or additional factor; 6. Assessment of outcome; 7. Follow-up long enough for outcomes to occur; 8. Adequacy of follow up of cohorts

### Data extraction

All available data from the identified studies were extracted respectively by two reviewers (Y.W and SW.L). If any disagreement achieved, a third reviewer (Y.T) would join in and reached a consensus. Extracted data were recorded in a standardized form including following items: first author’s surname, publication year, patients’ median or mean age, nationality, dominant ethnicity, number of patients, investigating method, cutoff value, follow-up time, and hazard ratios (HRs) for prognostic outcomes (overall survival [OS] and disease/recurrence/progression-free survival [DFS/RFS/PFS]) along with their 95% CI and p-values. Data were extracted from Kaplan–Meier curves to extrapolate HRs with 95% CIs by using previously described methods, when it could not be directly obtained from each article [16, 17]. Details of the aforementioned data were displayed in Tables 2 and 3.

**Table 2:** Main characteristics of studies included in this meta-analysis

First author	Publication year	Case nationality	Dominant ethnicity	Median or mean age	Study design	Malignant disease	Main type of pathology	Detected sample	Assay method	Survival analysis	Source of HR	Maximum months of follow-up
Chou [20]	2018	China	Asian	50	R	Glioblastoma multiforme	Glioma	Tissue	ihc	OS/PFS	Reported	207
Lu [10]	2017	China	Asian	60	R	Colorectal cancer	AdenoCa	Tissue	ihc	OS	SC	40
Huang [4]	2017	China	Asian	60	R	Colorectal cancer	AdenoCa	Tissue	ihc	OS	Reported	50
Li [11]	2017	China	Asian	40	R	Cervical carcinoma	SqCa	Tissue	ihc	OS	Reported	78
Shimura^a^ [41]	2017	Japan	Asian	55	R	Biliary cancer	AdenoCa	Serum	ELISA	OS	Reported	69.9
Shimura^b^ [41]	2017	Japan	Asian	55	R	Pancreatic cancer	AdenoCa	Serum	ELISA	OS	Reported	66
Wang [49]	2017	China	Asian	NM	R	Ovarian cancer	SqCa	Tissue	ihc	OS	Reported	72
Liu [37]	2017	China	Asian	65.1	R	Colorectal cancer	AdenoCa	Tissue	ihc	DFS	Reported	60
Gopalan [21]	2016	Australia	Caucasian	60	R	Colorectal cancer	AdenoCa	Tissue	ihc	OS	SC	110
Ilmer [12]	2016	American	Caucasian	47	R	Breast cancer	AdenoCa	Tissue	ihc	OS	SC	232
Yang [48]	2016	China	Asian	66.8	R	Colorectal cancer	AdenoCa	tissue	ihc	DFS	Reported	60
Tas [36]	2016	Turkey	Caucasian	59.5	R	Gastric cancer	AdenoCa	Serum	ELISA	OS	SC	97
Cheng [40]	2015	China	Asian	55.2	R	Gastric cancer	AdenoCa	Serum	ELISA	OS	SC	60
Lu [47]	2015	China	Asian	51	R	Ovarian cancer	SqCa	Tissue	ihc	OS	Reported	77
Jiang [22]	2014	China	Asian	50	R	Hepatocellular carcinoma	AdenoCa	Tissue	ihc	OS	Reported	87
Gomes [23]	2014	Brazil	Caucasian	50	R	Gastric cancer	AdenoCa	Tissue	ihc	OS	SC	55
Mu [44]	2013	China	Asian	66	R	Gastric cancer	AdenoCa	Tissue	ihc	OS	Reported	NM
Wu [45]	2013	China	Asian	59.6	R	Non-small cell lung cancer	SqCa	Tissue	ihc	OS	Reported	90
Liu [46]	2013	China	Asian	57.1	R	Non-small cell lung cancer	SqCa	Tissue	ihc	OS	SC	80
Yamaki [24]	2012	Japan	Asian	53	R	Breast cancer	AdenoCa	Tissue	ihc	OS/PFS	SC	13
Yang [25]	2012	China	Asian	45	R	Gallbladder carcinoma	AdenoCa	Tissue	ihc	OS	SC	18
Kim [26]	2012	Korea	Asian	60	R	Gastric cancer	AdenoCa	Tissue	ihc	OS	Reported	96
Kosacka [38]	2011	Poland	Caucasian	59.3	R	Non-small cell lung cancer	SqCa	Tissue	ihc	OS	SC	24
Povegliano [27]	2010	Brazil	Caucasian	50	R	Colorectal cancer	AdenoCa	Tissue	ihc	OS	SC	83
Canesin [42]	2010	American	Caucasian	NM	R	Bladder cancer	SqCa	Tissue	ihc	OS	SC	173
Vereecken [43]	2009	American	Caucasian	60	R	Melanoma	NM	Serum	ELISA	OS	Reported	60
Miranda [28]	2009	Brazil	Caucasian	59	R	Laryngeal carcinoma	SqCa	Tissue	ihc	DFS	SC	166
Szoke [29]	2007	German	Caucasian	58.8	R	Non-small cell lung cancer	SqCa	Tissue	ihc	OS	SC	127
Kang [30]	2007	Korea	Asian	63	R	Esophageal cancer	SqCa	Tissue	ihc	OS	SC	108
Moisa [39]	2007	Germany	Caucasian	56.8	R	Breast cancer	AdenoCa	Tissue	ihc	OS/DFS	Reported	185
Okada [13]	2006	Japan	Asian	63.9	R	Gastric cancer	AdenoCa	tissue	ihc	OS	Reported	72
Plzak [31]	2004	Prague	Caucasian	60	R	Head and neck carcinoma	SqCa	Tissue	ihc	OS	SC	60
Piantelli [14]	2002	Rome	Caucasian	60	R	Laryngeal carcinoma	SqCa	Tissue	ihc	OS/RFS	SC	90
Brule [32]	2000	Belgium	Caucasian	65	R	Prostate carcinomas	AdenoCa	Tissue	ihc	PFS	SC	86
Honjo [33]	2001	Japan	Asian	60	R	Tongue carcinoma	SqCa	Tissue	ihc	OS/DFS	SC	118
Nakamura [34]	1999	Japan	Asian	NM	R	Colorectal cancer	AdenoCa	Tissue	ihc	OS/DFS	SC	103
Sanjuan [35]	1997	Spain	Caucasian	NM	R	Colorectal cancer	AdenoCa	Tissue	ihc	OS/RFS	SC	96

*R* retrospective, *AdenoCa* adenocarcinoma, *SqCa* squamous carcinoma, *IHC* immunohistochemistry, *OS* overall survival, *DFS* disease-free survival, *PFS* progression-free survival, *RFS* recurrence-free survival, *SC* survival curve

^a, b^Data extracted from one study due to different malignant disease (biliary cancer and pancreatic cancer)

**Table 3:** HRs and 95% CIs of patient survival or cancer progression relating to galectin-3 expression in eligible studies

First author	Year	Malignant disease	Main type of pathology	Survival analysis	Cut off point	Case number		OS		DFS/RFS/PFS	
						High expression	Low expression	HR (95% CI)	p-value	HR (95% CI)	p-value
Chou [20]	2018	Glioblastoma multiforme	Glioma	OS/PFS	IRS score ≥ 2 (range 0–2)	NM	NM	1.34 (0.59–3.03)	0.478	0.181 (0.025–1.299)	0.089
Lu [10]	2017	Colorectal cancer	AdenoCa	OS	IRS score ≥ 2 (range 0–3)	43	14	1.88 (0.88–5.23)	0.0086	NM	NM
Huang [4]	2017	Colorectal cancer	AdenoCa	OS	IRS score ≥ 2 (range 0–4)	51	66	2.39 (1.12–4.75)	0.015	NM	NM
Li [11]	2017	Cervical carcinoma	SqCa	OS	IRS score ≥ 7 (range 0–12)	45	39	14.00 (1.75–112.31)	0.013	NM	NM
Shimura^a^ [41]	2017	Biliary cancer	AdenoCa	OS	≥ 10.3 ng/ml	22	2	6.19 (1.18–32.36)	0.031	NM	NM
Shimura^b^ [41]	2017	Pancreatic cancer	AdenoCa	OS	≥ 10.3 ng/ml	18	3	4.59 (1.17–17.68)	0.028	NM	NM
Wang [49]	2017	Ovarian cancer	SqCa	OS	30% of tumor cells stained	75	23	2.19 (1.17–4.02)	0.014	NM	NM
Liu [37]	2017	Colorectal cancer	AdenoCa	DFS	50% of tumor cells stained	38	23	NM	NM	2.10 (1.05–4.17)	< 0.05
Gopalan [21]	2016	Colorectal cancer	AdenoCa	OS	IRS score ≥ 3 (range 0–4)	69	4	4.00 (0.90–20.00)	0.052	NM	NM
Ilmer [12]	2016	Breast cancer	AdenoCa	OS	Hscore ≥ 150	23	64	0.69 (0.17–2.86)	0.019	NM	NM
Yang [48]	2016	Colorectal cancer	AdenoCa	DFS	IRS score ≥ 4	40	24	NM	NM	2.09 (1.09–3.79)	< 0.05
Tas [36]	2016	Gastric cancer	AdenoCa	OS	NM	29	29	0.79 (0.37–1.67)	0.54	NM	NM
Cheng [40]	2015	Gastric cancer	AdenoCa	OS	≥ 16.4 ng/ml	43	43	1.63 (0.72–3.66)	0.099	NM	NM
Lu [47]	2015	Ovarian cancer	SqCa	OS	IRS score ≥ 5	23	54	2.32 (1.05–5.10)	0.036	NM	NM
Jiang [22]	2014	Hepatocellular carcinoma	AdenoCa	OS	IRS score ≥ 4	135	30	7.51 (3.00–18.78)	< 0.01	NM	NM
Gomes [23]	2014	Gastric cancer	AdenoCa	OS	50% of tumor cells stained	31	26	0.73 (0.27–1.98)	0.798	NM	NM
Mu [44]	2013	Gastric cancer	AdenoCa	OS	≥ 10.0 ng/ml	NM	NM	1.58 (1.11–2.86)	0.013	NM	NM
Wu [45]	2013	Non-small cell lung cancer	SqCa	OS	IRS score ≥ 2 (range 0–2)	102	58	2.05 (1.15–3.67)	0.015	NM	NM
Liu [46]	2013	Non-small cell lung cancer	SqCa	OS	10% of tumor cells stained	52	10	3.09 (1.23–5.26)	0.045	NM	NM
Yamaki [24]	2012	Breast cancer	AdenoCa	OS/PFS	30% of tumor cells stained	67	49	0.90 (0.15–5.35)	0.041	0.46 (0.18–1.22)	0.018
Yang [25]	2012	Gallbladder carcinoma	AdenoCa	OS	25% of tumor cells stained	67	41	1.68 (1.05–2.69)	0.028	NM	NM
Kim [26]	2012	Gastric cancer	AdenoCa	OS	10% of tumor cells stained	397	74	0.80 (0.51–1.26)	0.331	NM	NM
Kosacka [38]	2011	Non-small cell lung cancer	SqCa	OS	10% of tumor cells stained	18	29	1.24 (0.38–4.05)	0.84	NM	NM
Povegliano [27]	2010	Colorectal cancer	AdenoCa	OS	50% of tumor cells stained	32	43	1.28 (0.01–138.79)	0.056	NM	NM
Canesin [42]	2010	Bladder cancer	SqCa	OS	20% of tumor cells stained	194	194	2.34 (1.81–3.02)	< 0.001	NM	NM
Vereecken [43]	2009	Melanoma	NM	OS	≥ 10.0 ng/ml	NM	NM	4.64 (2.17–9.91)	0.0001	NM	NM
Miranda [28]	2009	Laryngeal carcinoma	SqCa	DFS	NM	47	18	NM	NM	1.06 (0.44–2.60)	0.5284
Szoke [29]	2007	Non-small cell lung cancer	SqCa	OS	NM	51	41	1.86 (1.09–3.15)	0.003	NM	NM
Kang [30]	2007	Esophageal cancer	SqCa	OS	IRS score ≥ 2 (range 0–4)	18	44	0.98 (0.56–1.70)	0.227	NM	NM
Moisa [39]	2007	Breast cancer	AdenoCa	OS/DFS	IRS score ≥ 2 (range 0–3)	52	146	1.41 (1.16–3.89)	0.013	1.65 (0.91–2.87)	0.09
Okada [13]	2006	Gastric cancer	AdenoCa	OS	60% of tumor cells stained	60	55	0.26 (0.11–0.64)	0.0031	NM	NM
Plzak [31]	2004	Head and neck carcinoma	SqCa	OS	50% of tumor cells stained	23	30	0.30 (0.06–1.64)	0.0024	NM	NM
Piantelli [14]	2002	Laryngeal carcinoma	SqCa	OS/RFS	5% of tumor cells stained	42	31	0.54 (0.13–2.23)	0.0001	0.49 (0.20–1.21)	0.0013
Brule [32]	2000	Prostate carcinomas	AdenoCa	PFS	IRS score ≥ 1.5 (range 0–2)	25	102	NM	NM	3.45 (1.49–7.95)	0.044
Honjo [33]	2001	Tongue carcinoma	SqCa	OS/DFS	85% of tumor cells stained	31	23	3.51 (1.32–9.37)	0.012	2.30 (0.83–6.33)	0.021
Nakamura [34]	1999	Colorectal cancer	AdenoCa	OS/DFS	66.7% of tumor cells stained	36	71	3.63 (1.88–7.01)	0.014	2.65 (1.54–4.58)	0.0224
Sanjuan [35]	1997	Colorectal cancer	AdenoCa	OS/RFS	25% of tumor cells stained	83	68	4.15 (2.01–8.55)	0.0086	3.32 (1.67–6.60)	0.01

*AdenoCa* adenocarcinoma, *SqCa* squamous carcinoma, *OS* overall survival, *DFS* disease-free survival, *PFS* progression-free survival, *RFS* recurrence-free survival, *NM* not mentioned, *IRS* immunoreactivity score, *Hscore* the intensity and respective percentage cells that stain at each intensity were multiplied to reach a Hscore that ranged from 0 to 300, *OS* overall survival, *HR* hazard ratio, *CI* confidence interval

^a, b^Data extracted from one study due to different malignant disease (biliary cancer and pancreatic cancer)

### Statistical analysis

Based on available data, the relationship between galectin-3 and multiple human malignant neoplasms was conducted by OS or DFS/RFS/PFS and the pooled hazard ratios (HRs) with 95% confidence intervals (CIs) were utilized to evaluate their efficacy. The effect of heterogeneity was quantified via *I*^2^= 100% × (Q − df)/Q. If significant heterogeneity (*p *< *0.1* or *I*^2^> 50%) existed, the random-effects model (DerSimonian–Laird method) would be applied; otherwise, a fixed-effects model (Mantel–Haenszel method) would be utilized [18]. Moreover, in the case of significant heterogeneity, subgroup analysis was carried out by the type of malignant disease and dominant ethnicity to further minimize the influence. Sensitivity analysis was conducted to access the stability of results by deleting one single study each time to reflect the impact of the individual to overall. Publication bias was evaluated by the Begg’s funnel plot and Egger linear regression test with a funnel plot [19]. If *p *< 0.05, it indicated the existence of publication bias. All p-values were calculated using a two-sided test and *p *< 0.05 was considered statistically significant. Besides, all statistical data were conducted by Stata software (version 12.0; StataCorp LP, College Station, TX) and Microsoft Excel (V.2007, Microsoft Corporation, Redmond, WA, USA).

## Results

### Summary of enrolled studies

**Fig. 1:**
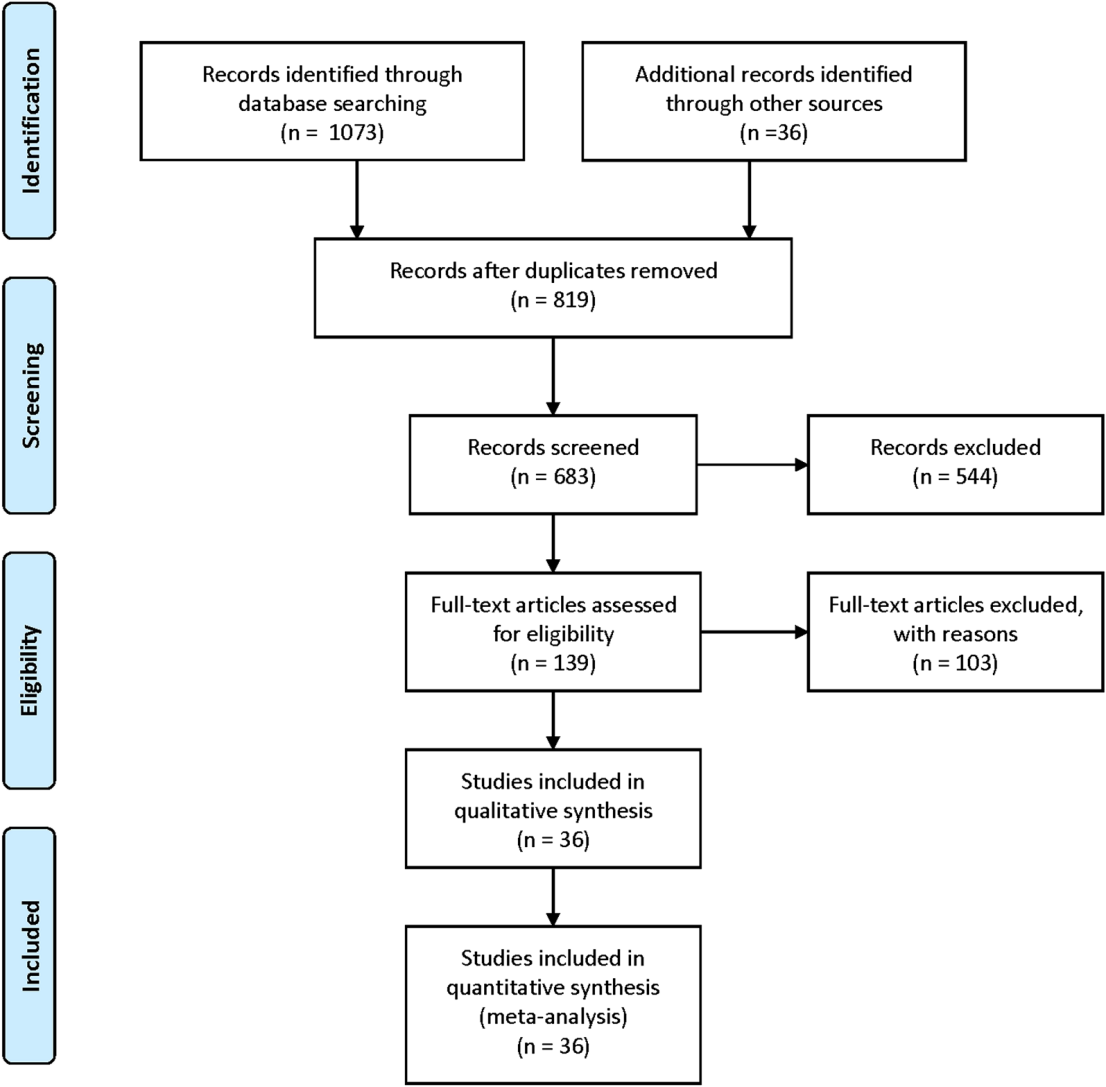
Flow diagram of the literature selection process

The literature search yielded 1109 citations through online databases by means of previous search strategy. Amongst them, 970 records were excluded because of reviews, letters, case-reports, duplicates and so on, after screening the tittles and abstracts. The full texts of the remaining 139 articles were evaluated by the reviewers. Among them, 103 potentially suitable studies were excluded because of lacking sufficient survival data (HRs and 95% CIs), not related to OS or DFS/RFS/PFS, absence of key information. Ultimately, 36 studies were considered to be eligible for this meta-analysis (Fig. 1) [4, 10–14, 20–49].

Detailed quality assessments of each eligible article were presented in Table 1 and the main characteristics of these 36 enrolled studies were summarized in Tables 2 and 3. Amongst them, 33 studies focused on OS and 11 articles investigated DFS or PFS or RFS. 15 of these records focused on Caucasian populations, which mainly came from European countries, and 22 focused on Asian populations. As for cancer type, malignant neoplasms assessed in this article included colorectal carcinoma, gastric carcinoma, breast cancer, laryngeal squamous cell carcinoma (LSCC), esophageal squamous cell carcinoma (ESCC), glioblastoma multiforme, cervical carcinoma, hepatocellular carcinoma, gallbladder carcinoma, non-small cell lung cancer, head and neck carcinoma, prostate carcinomas, tongue carcinoma, biliary cancer, pancreatic cancer, ovarian cancer, bladder cancer and melanoma. Besides, all these aforementioned studies were retrospective.

### OS associated with galectin-3 expression

**Fig. 2:**
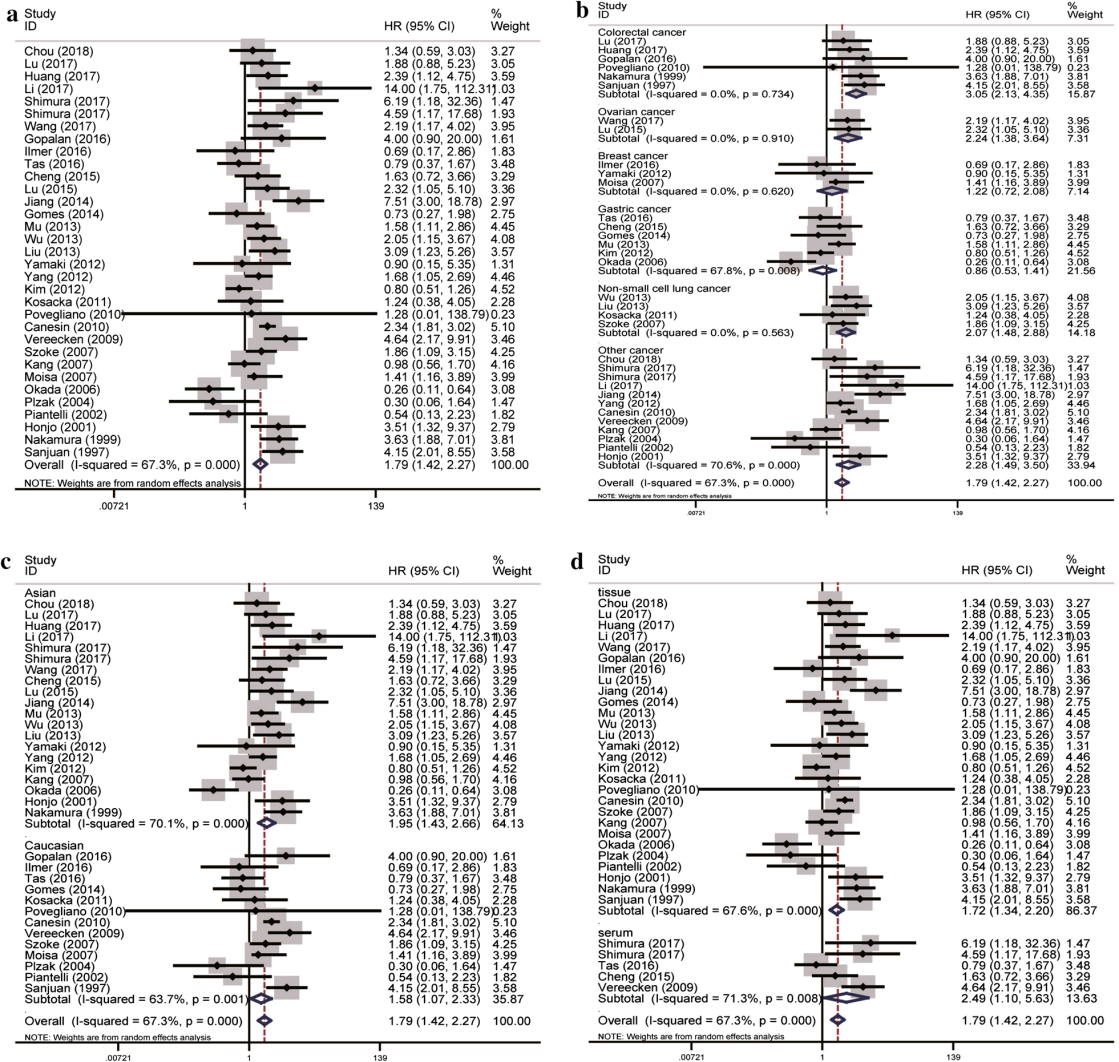
Forest plots of OS in association with galectin-3 in various cancers. **a** The overall group; **b** the subgroup analysis of cancer types; **c** the subgroup analysis of dominant ethnicity; **d** the subgroup analysis of detected samples

A total of 33 eligible studies were enrolled to evaluate the role of elevated galectin-3 expression in multiple human malignant neoplasms by OS, within a random-effects model. Our results did indicate that high galectin-3 expression was significantly associated with unfavorable OS in overall cancer patients (pooled HR = 1.79, 95% CI 1.42–2.27, *I*^2^= 67.3%, *p* < 0.01; Fig. 2a). In the subgroup analysis of specific cancer type, we found high expression of galectin-3 correlated with reduced OS in colorectal cancer, ovarian cancer and non-small cell lung cancer (pooled HR = 3.05, 95% CI 2.13–4.35, *I*^2^= 0.0%, *p *= 0.734; pooled HR = 2.24, 95% CI 1.38–3.64, *I*^2^= 0.0%, *p *= 0.910; pooled HR = 2.07, 95% CI 1.48–2.88, *I*^2^= 0.0%, *p *= 0.563; respectively) (Fig. 2b). Furthermore, in terms of dominant ethnicity subgroup analysis, both the Asian and Caucasian ethnicity were statistically significant (pooled HR = 1.95, 95% CI 1.43–2.66, *I*^2^= 70.1%, *p *< 0.01; pooled HR = 1.58, 95% CI 1.07–2.33, *I*^2^= 63.7%, *p *= 0.001; separately) (Fig. 2c). Besides, no matter galectin-3 in the tissue or in the plasma, its elevated expression was associated with reduced OS (pooled HR = 1.72, 95% CI 1.34–2.20, *I*^2^= 67.6%, p < 0.01; pooled HR = 2.49, 95% CI 1.10–5.63, *I*^2^= 71.3%, p = 0.008; respectively) (Fig. 2d).

### DFS/RFS/PFS associated with galectin-3 expression

**Fig. 3:**
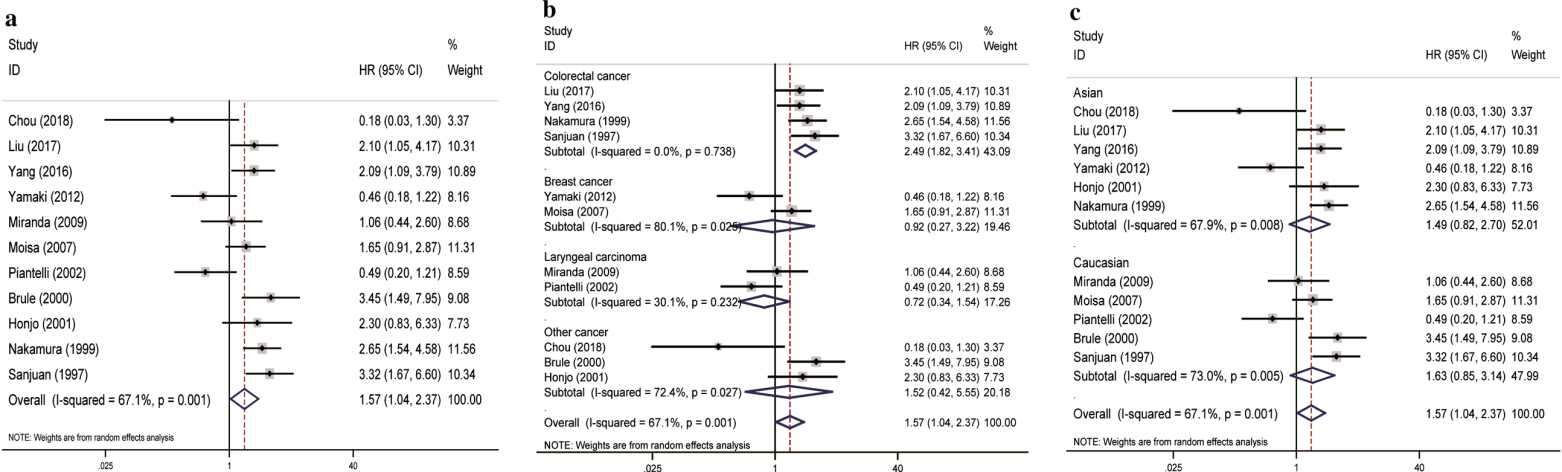
Forest plots of DFS/RFS/PFS in association with galectin-3 in various cancers. **a** The overall group; **b** the subgroup analysis of cancer types; **c** the subgroup analysis of dominant ethnicity

A total of 11 original studies were included to evaluate the role of elevated galectin-3 expression in patients with various solid tumors by DFS/RFS/PFS, within a random-effects model. Our results successfully identified the significant association of high galectin-3 expression with reduced DFS/RFS/PFS in overall cancer patients (pooled HR = 1.57, 95% CI 1.04–2.37, *I*^2^= 67.1%, *p *= 0.001; Fig. 3a). In the subgroup analysis of specific cancer type, we found that high expression of galectin-3 was correlated with shorter DFS/RFS/PFS in colorectal cancer (pooled HR = 2.49, 95% CI 1.82–3.41, *I*^2^ = 0.0%, p = 0.738; Fig. 3b). However, In terms of dominant ethnicity subgroup analysis, both the Asian and Caucasian ethnicity were not statistically significant (Fig. 3c).

### Sensitivity analyses

**Fig. 4:**
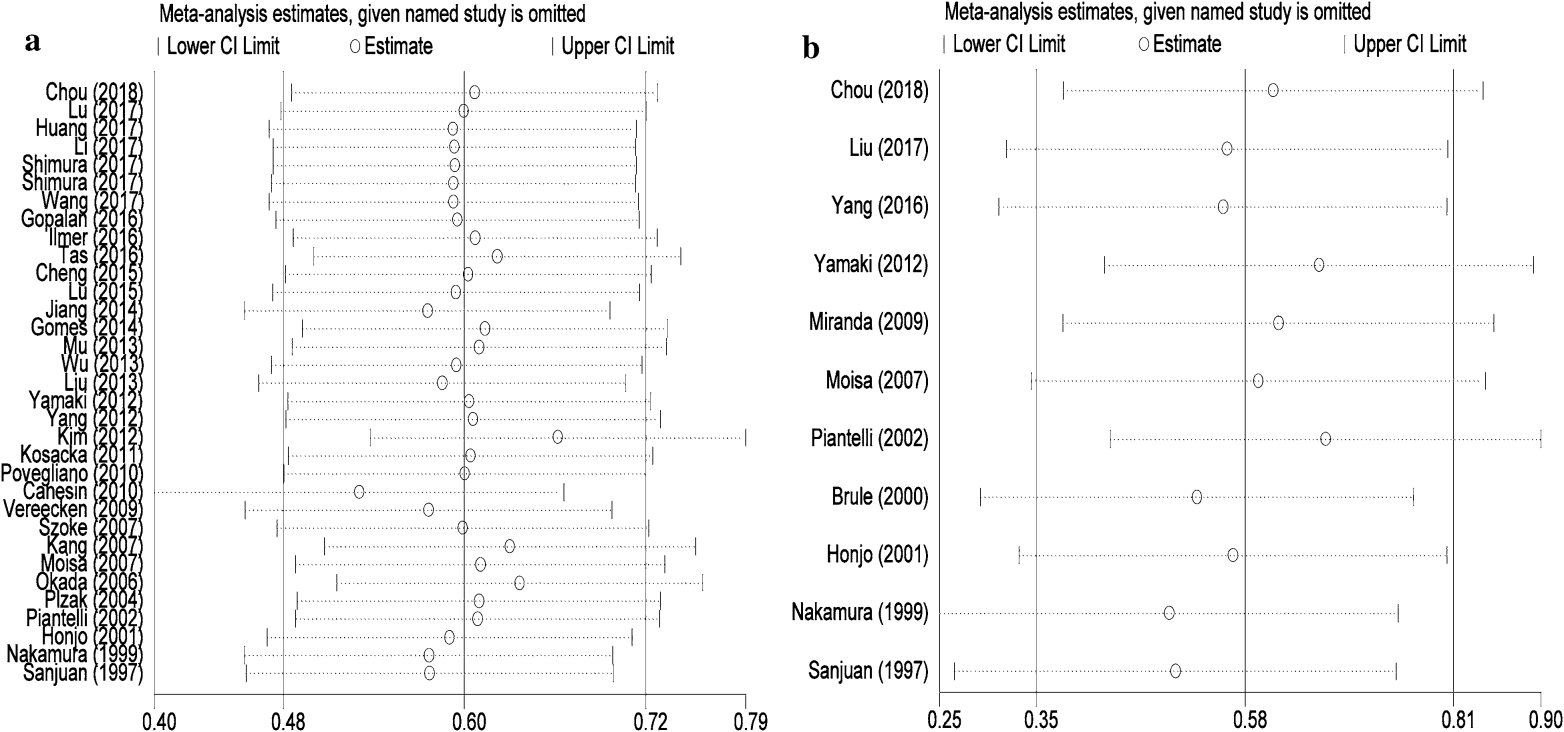
Sensitivity analysis of each included study. **a** OS for individual studies. **b** DFS/RFS/PFS for individual studies

In order to determine the robustness and the stability of our results, sensitivity analysis was conducted to access the stability of results by deleting one single study each time, to reflect the impact of the individual to overall. Our results indicated that no single study significantly influenced the pooled OR and 95% CIs. Namely, our results are comparatively reliable and stable (Fig. 4).

### Publication bias

**Fig. 5:**
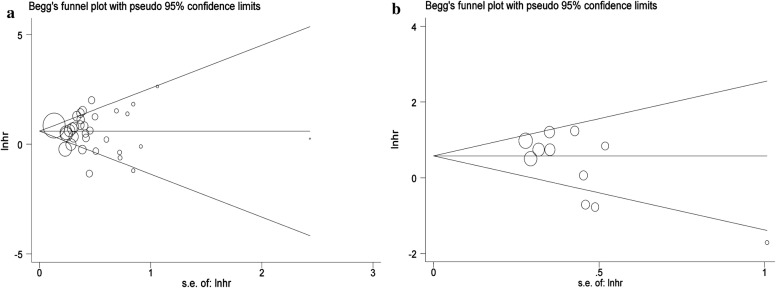
Begg’s funnel plots of the publication bias. **a** OS for individual studies. **b** DFS/RFS/PFS for individual studies

The combined application of Begg’s and Egger’s test was utilized to evaluate the publication bias and meanwhile the funnel plots were displayed in Fig. 5. In the pooled analysis of OS or DFS/RFS/PFS, the *p* values of Begg’s test and the *p* values of Egger’s test were all above 0.05, indicating no publication bias in this study.

## Discussion

Up to now, elaborate efforts have been made to establish reliable and convincing evidence to detect promising biomarkers for patients with solid tumors. Galectins, as a family of animal carbohydrate-binding proteins, which had the ability to agglutinate cells, were considered to be potential biomarkers of cancer prognosis given their unique structure and functions into consideration [50, 51]. Over the past years, galectins have been implicated in the development of cancer, the pathogenesis of heart failure and ventricular remodeling, infectious processes, and inflammatory processes [52]. Amongst them, due to its differential expression between cancer and normal tissues, galectin-3 was regarded as one important member of galectins family. However, the definite role of galectin-3 in various human malignant neoplasms remained inconsistent. Hence, this meta-analysis was conducted to clarify this question.

It was the first time for us to shed light on the association between elevated galectin-3 expression and the prognosis of patients with solid tumors. Meanwhile, our results were the systematic evaluation of the prognostic outcomes (OS or DFS/RFS/PFS) in a larger population. Our results did suggest that galectin-3 play an oncogenic role in overall cancer patients. Moreover, we found that high expression of galectin-3 was correlated with shorter OS or DFS/RFS/PFS in colorectal cancer and meanwhile it merely associated with reduced OS in ovarian cancer or non-small cell lung cancer, indicating that it could be a promising biomarker and a novel therapeutic target for them. Furthermore, in subgroup analyses of dominant ethnicity, we observed that both the Asian and Caucasian ethnicity were statistically significant for OS, suggesting that the detection of high galectin-3 expression in these patients might be useful for prognosis prediction. Besides, the outcomes of us shed light on that no matter galectin-3 in the tissue or in the plasma, its role remained stable, indicating it could be a promising biomarker and a novel therapeutic target. Meanwhile, according to the results of sensitivity analyses and publication bias, no single study significantly influenced the pooled OR and 95% CIs and no obvious publication bias was detected in this meta-analysis, indicating the robustness and the stability of our results.

Previous researches indicated that increased expression of galectin-3 often predicted unfavorable outcomes and the level of galectin-3 was positively correlated with invasion of depth, vessel invasion, lymph node metastasis, distant metastasis, and TNM stages of various cancers [26, 53]. Tao et al. [37] demonstrated that the positive expression of galectin-3 was associated with more malignant biological behavior of colorectal cancer and it could be used as a predictor of poor prognosis for patients. As for tongue carcinomas, Honjo showed that cytoplasmic galectin-3 expression increased during the progression from normal to cancerous states, whereas nuclear galectin-3 expression decreased during the progression from normal to cancerous states, indicating that enhanced expression of cytoplasmic galectin-3 could serve as a predictor of disease recurrence in these patients [33].

As for its relevant mechanisms, several studies found that galectin-3 was expressed in both cytosol and nucleus [10, 54]. Therein as an important regulator of the Wnt/β-catenin signaling pathway, galectin-3 could activate the epithelial–mesenchymal transition (EMT) in tumor cells to promote the invasion and metastasis of cancer [55, 56]. Furthermore, it could subsequently activate the Ras-mediated Akt signaling pathway to inhibit cell apoptosis by interacting with the activated GTP-bound K-Ras [57]. Besides, it could also modulate VEGF- and bFGF-mediated angiogenesis by binding its carbohydrate recognition domains (CRDs) to integrate αvβ3, and then promote the growth of new blood vessels [58].

As for the effects on heterogeneity, subgroup analysis was a way to discover their potential sources and even decrease the huge heterogeneity. As presented by our results, we could easily find that there might be the existence of significant heterogeneity of elevated galectin-3 expression in the overall cancer patients. So we conducted a subgroup analysis based on the specific cancer types and found that most of their heterogeneity decreased significantly, even with no heterogeneity. However, subgroup analysis of dominant ethnicity was not associated with significant reduction of heterogeneity, indicating that the dominating source of heterogeneity might be the different cancer types.

Sometimes, galectin-3 combined with another biomarker was often utilized simultaneously in prognostic outcome analyses, showing it might not be an independent factor affecting the prognosis of cancer patients. As indicated by Li et al. [11] the expressions of ezrin and galectin-3 were correlated with the development of cervical cancer, and over-expressions of those proteins were indicative of poor prognosis in patients with cervical cancer. Galectin-3 associated with cyclin D1 expression was also studied in non-small cell lung cancer. As a result, no important correlations with clinicopathological findings and no prognostic values were revealed between them. However, higher cyclin D1 expression was found in galectin-3 negative tumor tissues and the differences in correlations between their expressions in two main histopathological types of non-small cell lung cancer were also discovered [38].

The strength of this study was our broad search strategy with few restrictions to minimize any potential publication bias. Moreover, this was the first meta-analysis reporting the prognostic value of galectin-3 for cancers in the medical literature, which could provide some references for clinical work. Although this meta-analysis was performed with rigorous statistics, our conclusion still had several limitations for the following reasons. Firstly, different studies had their own varied expression cut-off values, which brought many difficulties for us to define the standard cutoff value, resulting in bias in the results of the effectiveness of galectin-3 as a prognostic factor in cancer patients. Secondly, heterogeneity existed in the total OS and DFS/RFS/PFS group and it was likely due to the different characteristics of the patients, such as the age, cancer type, different method in detecting samples and the varied cut-off values of galectin-3 expression. Thirdly, due to the insufficient studies, correlation between galectin-3 and OS or DFS/RFS/PFS in other tumor types has not been further analyzed. Fourthly, some essays studied galectin-3 combined with another biomarker in prognostic outcome analyses, showing galectin-3 was not an independent factor affecting the prognosis of cancer patients. Last but not least, all of these enrolled studies were derived from retrospective or observational data, which could not have a clear impact on group baseline features as RCTs. Upcoming prospective RCTs were required to provide more available data. Taking these aforementioned limitations into consideration, our results could be interpreted rigorously and meanwhile more well-designed studies were required to verify our findings.

## Conclusions

In summary, it was the first time for us to shed light on the prognostic role of elevated galectin-3 expression in various cancers. Our results did suggest that galectin-3 played an oncogenic role in colorectal cancer, ovarian cancer and non-small cell lung cancer, indicating that it could be a promising biomarker for predicting the prognosis of patients with malignant neoplasms, and the biological functions of galectin-3 were of great research value of the subject. Due to the aforementioned limitations, larger samples of more strictly designed studies were required to provide more high-quality data to elaborate their associations.
